# 

**Published:** 1906-11-15

**Authors:** 


### ANNOUNCEMENTS.

IOWA STATE BOARD OF EXAMINERS—The meeting of the Iowa State Board of Dental Examiners for December 4th has been postponed until a later date.—E. D. Brower, *Secy.*

BANQUET TO DR. G. V. BLACK—The Fraternal Dental Society and the St. Louis Society of Dental Science, will unite in giving a banquet in honor of Dr. G. V. Black, at the Jefferson Hotel, St. Louis. The evening of January 15th, 1907.

The afternoon of the same day Dr. Black will deliver an illustrated lecture on some phase of Operative Dentistry.

The long and untiring efforts and valuable scientific contributions of Dr. Black easily makes him the foremost dental scientist the world has ever produced. No dentist living or dead, so much deserves the thanks and praise of his professional associates. A most cordial invitation is extended to the members of the profession to be present at both lecture and banquet and assist in honoring Dr. Black. Those desiring covers reserved for banquet will remit $5.00, price per plate, to Dr. Richard Summa, *Sec'y.*, Oriel Building, St. Louis, before January 12th.

*Committee*—Geo. A. Bowman, *Chairman*, A. H. Fuller, Edward H. Angle, D. O. M. LeCron, Adam Flickinger, Wm. Conrad, Burton Lee Thorpe.

—✦—

INSTITUTE OF DENTAL PEDAGOGICS.—The Fourteenth Annual Meeting of the Institute of Dental Pedagogics will be held in Chicago on the 27, 28, and 29th of December.

*The following subjects will be discussed*—Teaching of Anesthesia: the Emergencies, how treated how prevented. Teaching of Operative Technic. Teaching of Prosthetic Technic. A Method of teaching Orthodontia. Teaching Porcelain Technic. Teaching of Materia Medica. Report of Committee on Dental Nomenclature.—W. Earl Willmott, *Sec'y.*

—✦—

OHIO STATE DENTAL SOCIETY.—The Forty-First Annual Meeting of The Ohio State Dental Society will be held in Columbus December 4, 5, and 6, 1906.

A program of varied and exceptional interest has been provided and we even hope to surpass the very successful meetings of recent years. The papers are of the best, the clinics numerous and instructive, the exhibits extensive and complete.

The membership of this society has nearly doubled in the last three years.

If you are not a member and are eligible *come and join*, *but—COME*

J. R. Chapman, *Sec'y.*

---

THE KENTUCKY STATE BOARD OF DENTAL EXAMINERS—Will hold their next meeting for the examination of applicants, the First Tuesday in December. Every applicant must be a graduate of a reputable Dental College.

For further information address the President of the Board.—J. Richard Wallace, "The Masonic", Louisville, Ky.

---

*To the Editor—*

The parliamentary status of the Army and Navy Dental Bills continues unchanged from the past to the next session of the 59th Congress and, therefore, either bill may be called from the House Calendar and passed as a separate measure or added as an amendent to any subject of legislation to which it may be germane.

The Navy Bill, H. R.—13851, provides for 30 individual dental surgeons with "the rank and compensation of Acting Assistant Surgeon," but falls short of what was sought by the N. D. A. Committee in that it fails to provide corps organization, regular rank with right to promotion and retired pay, and dental examiners to examine in dental subjects. How ever, this bill has had the approval of the N. D. A. Committee since January, 1905, as the best obtainable and because of an understanding that the enactment of the Army Bill would establish a precedent for a substantially identical measure for the Navy. It was also understood that the parliamentary status attained by a favorable report on H. R.—13851, with its provision for limited ranks, would be advantageous in promoting Senate 3150 with its provision for regular rank.

The law under which Thirty Army Dental Surgeons are now employed was framed and promoted to its passage by the N. D. A. Committee, under instructions recommended by Drs. McManus, Hinman and Noel, *to wit;*

"In reference to the report of Drs. Donnally and Finley, we do not entirely agree with them 'that we should work for status only', but recommend that this Association work for the admission of dentsits into the Army and Navy and then for their status". Accordingly the passage of the contract bill, February 2nd, 1901, was followed at the next session of Congress by Army and Navy Dental Bills identical in their provisions for corps organization, grades of rank, and eligibility requirements, the Army Bill differing in that it made contract dental surgeons eligible to rank regardless of age and without tests of physical or professional fitness. The Army Bill was then favored by Surgeon Sternberg, but vigorously and successfully opposed by Secretary Root, and thereafter was opposed by the Surgeon General, the General Staff, the Chief of Staff and Secretary Taft, an array of opposing military authority against which the resources of the N. D. A. Committee made slow though equal progress in Congress with the medical bill which is advocated by all these authorities, by President Roosevelt and by the united medical profession speaking through its national body, 38 state and 2300 county societies and many of its colleges and individual members.

The first tangible step in the legislative progress of the pending Army Dental Bill was when the Surgeon General yielding in a measure to the N. D. A. Committee, agreed to favor a bill to be drawn in accord with the views he expressed officially under date of April 15th, 1904. His views, briefly stated were:

That the number of dental surgeons should not exceed

30: That all should start with the rank of Lieutenant: That all alike should be required to meet prescribed age, physical and professional standards; That on examination, 5 should be promoted to Captain after 5 years and 3 to Major after 15 years service: and that the dental examining board should be composed of one dental surgeon selected by the Secretary of War from the contract dental surgeons and two dental surgeons nominated to the Secretary of War by the President of the American Dental Association".

No bill to exactly accord with these views was framed, but, taking these views as an only basis for a compromise, the provisions of Senate 2355 were favored and presented to Congress January 26, 1905, with the all important approval of the Surgeon General and the all but fatal renewed disapproval of the General Staff and Chief of Staff.

The more important consession contained in this bill which were not included in the official views of April 15, 1904, are:

(*a*) An increase of about 100 per-cent in the number of dental surgeons.

(*b*) An unprecedent exemption of all contract dental surgeons from an age standard universally required of commissioned officers.

(*c*) An unparallelled liberality in accepting, at the option of the Surgeon General the entrance contract physical and professional examinations instead of a re-examination on the higher standard required for commissioned rank—a concession not granted or proposed to be granted the contract surgeons.

(*d*) Granting mounted rank and reckoning time served in contract positions in computing longevity pay, thus at once increasing this pay from $260 to $400 per annum.

(*e*) Creating 3 Captaincies instead of them being earned by 5 years service and making them eligible to majorities after 10 years instead of the 15 years service, and increasing the whole number of Captains 3 fold and the majors 2 fold.

(*f*) Changing the Surgeons General recommendation that two examiners be "nominated to the Secretary of War by the President of the American Dental Association" to certification of their qualification by the ExecutiveCouncil of the N. D. A. thus changing the "nomination" by one man to certification of qualification" by seven men and changing the appointing power from a political to a medical officer.

With these liberal provisions, which were not all desired though more than could reasonably be expected to escape Congressmen who were prompted to give them special scrutiny,the bill passed the Senate April 23rd, but with an ill-ad vised amendant by which it was intended to displace the board provision by one which would place an Army Surgeon at the head of a board to examine in practical dentistry and the subjects of a dental college course.

If this amendent should prevail against the recommendation of the Surgeon General the N. D. A. Committee and the Senate and House Military Committees, and the view expressed by the N. D. A. in 1900 on the recommendation of Drs. C. N. Johnson, Jas. McManus and T. T. Moore, *to wit;*

"We respectflly ask the Association to carefully consider the question of the Surgeon General's intimation that this Association suggest candidates for appointment as examining dental surgeons under the proposed law, and the Committee feels that the power to recommend resides with the Executive Council". it is believed by the best informed that it will create friction between medical and dental officers, give undue weight to the surgeon's estimate of the value of the dental educational course, deprive the N. D. A. of intended recognition of its philanthropic motives in successfully promoting an Army Bill of humane and economic value,and, in seeking further legislation this surgeon's views, rather than the dental surgeon's, would likely prevail with the War Department authorities. It would also deprive the Surgeon General of the right to retain in the service, as a civilian examiner, any professionally qualified contract dental surgeon who might, through the amendments or by reason of age or physical condtion, fail to secure regular rank.

On April 28th, the House Military Committee reported favorably this bill and recommended the adoption of amendments restoring the original board provision, limiting the number of dental surgeons to 45, requiring one year's probation under contract as a test of fitness for rank, and requiring that all contract dental surgeons, in order to be eligible for rank, must have been within the standard age (30 years) at the time they entered the contract service.

These proposed amendments should not have been made an occasion for ill considered comment, or for the use of means to restore the original provisions of the bill, that would reflect on the good faith and just intent of the Military Committee or other committee or person. More objectionable provisions had been proposed and through the quiet and orderly effort of the N. D. A. Committee withdrawn. It is true that under one of the proposed amendments, (*if adopted without modification*) seven contract dental surgeons would lose their positions, but, through the immediate co-operation of members of the Military Committee, the Surgeon General and the N. D. A. Committee the modification of the effect of this amendment was assured within a fewdays after it was proposed.

In order to make sure of the retention in the service of *all* the seven over-aged without loss of pay tenure or prospect of more favorable legislation later, it is necessary that they and their friends should feel and show an equal interest in each of the seven, (that is, for the seven as a class) and not seek a personal advantage for one over another. Under such a condition the plans of the Surgeons General and the N. D. A Committee may be safely trusted to carry, without loss to any one, while all that has been gained through strenuous effect for the profession and for the Army and Navy Corps may be retained and enlarged with enduring honor and increasing benefit to the profession.

Yours faithfully,

Wms. Donnally.

*September 4th,* 1906.

